# Squamous cell carcinoma in kidney with chronic pyelonephritis and pyelonephrosis: a rare case

**DOI:** 10.11604/pamj.2023.45.31.39117

**Published:** 2023-05-15

**Authors:** Pragyamita Datta

**Affiliations:** 1 Department of Pathology, Jawaharlal Nehru Medical College (DMIHER), Sawangi, Wardha, Maharashtra, India

**Keywords:** Calculus, kidney, pyelonephritis, squamous cell carcinoma

## Image in medicine

Primary squamous cell carcinoma contributes around 0.5 to 15% of all urothelial malignancy. The predisposing factor and causative agents include renal calculi, infection and endogenous and exogenous chemicals, hormonal imbalance and vitamin A deficiency. A 56-year-old female presented with right flank pain on and off with fever for the last one-week duration, imaging study revealed dilated calyx containing pus material, one focus showing a small whitish nodule. On gross examination, there was a complete loss of architecture, and one portion of calyx showed a friable whitish area and one small nodule measuring 1 x 0.5 cm. Microscopically showing an irregular nest with sheets of malignant squamous cells, differentiating into keratin pearls and background is traversed stromal invasion.

**Figure 1:**
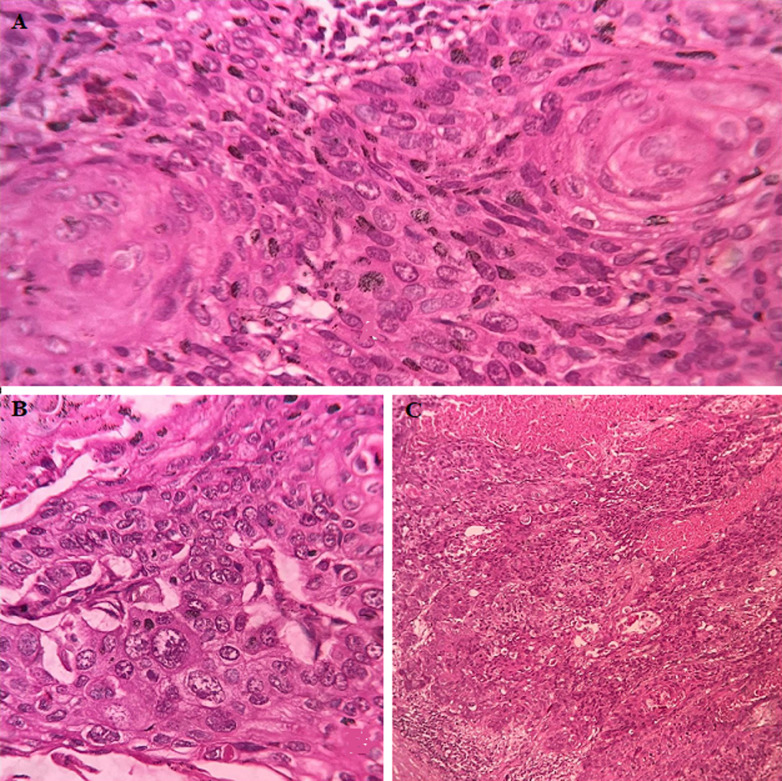
A, B, C) microscopically showing irregular nest and malignant cells arranged in sheet; squamous differentiation showing keratin pearls and background is traversed by stromal invasion

